# Prevalence and characteristics of chronic Pain in the Chinese community-dwelling elderly: a cross-sectional study

**DOI:** 10.1186/s12877-021-02432-2

**Published:** 2021-10-07

**Authors:** Xiahui Li, Wei Zhu, Jiping Li, Chan Huang, Fan Yang

**Affiliations:** 1 West China School of Nursing, Sichuan University, Chengdu City, Sichuan Province China; 2 West China Hospital, Sichuan University, Chengdu City, 610041 Sichuan Province China; 3 School of Nursing, Chengdu University, Chengdu City, Sichuan Province China; 4 West China Second Hospital, Sichuan University, Chengdu City, Sichuan Province China

**Keywords:** Chronic pain, The elderly, Community-dwelling, Cross-sectional study

## Abstract

**Background:**

Chronic pain adversely affects health and daily life in the elderly. Gaining insight into chronic pain that affects the community-dwelling elderly is crucial for pain management in China, which possesses the largest elderly population in the world.

**Methods:**

This is a cross-sectional design study that followed the STROBE Guideline. A randomized cluster sampling method was used to recruit participants in the Sichuan Province from Dec 2018 to May 2019. In addition, face-to-face interviews were conducted to collect socio-demographic data, characteristics and health-seeking behaviors of chronic pain through a self-designed questionnaire.

**Results:**

A total of 1381 older adults participated in this study. Among these participants, 791 (57.3%) had chronic pain. Here, prevalence and pain intensity were both found to increase from the 60–69 group to the 70–79 group, which then decreased in the ≥80 group with no significant differences in sex (*p* > 0.05). The most common pain locations were observed in the legs/feet (53.5%), head (23.6) and abdomen/pelvis (21.1%). Among the elderly suffering from chronic pain, 29.4% sought medical help, 59.2% received medication and 59.7% adopted non-drug therapy.

**Conclusion:**

Chronic pain is a common health concern in the Chinese community-dwelling elderly, which possesses different characteristics than other countries’ populations. Therefore, easier access to medication assistance and provision of scientific guidance for non-drug therapy may serve as satisfactory approaches in improving pain management.

**Supplementary Information:**

The online version contains supplementary material available at 10.1186/s12877-021-02432-2.

## Background

The International Association for the Study of Pain (IASP) has defined chronic pain as an unpleasant sensory and emotional experience may be associated with tissue damage, which may also be described in term of such damage lasting for over 3 months [1]. With the population aging, a rise in prevalence for chronic and degenerated diseases is inevitable, leading to a high incidence of chronic pain in the elderly [2, 3]. Previous studies have investigated the incidence of chronic pain in the elderly, showing a prevalence ranging from 43.8 to 55.2% in elderly population residing in the east [4–7] while 40 to 66% prevalent among those in the west [8, 9]. Moreover, the most common sites of pain in older people are the back, leg, knee or hip, and ‘other’ joints (such as joints of fingers, wrists and toes) in the most developed countries [10].

Chronic pain significantly impairs the health status and daily life of the elderly. Previous studies have found that chronic pain impairs the activities of daily living [11, 12], dignity [13], sleep quality [14] and quality of life [15]. Chronic pain was also reported to cause social isolation, fatigue and depression in the elderly [8, 16]. Moreover, chronic pain can increase the utilization of medical resources of society as well as the healthcare costs of individuals [15, 17]. Despite a series of studies on chronic pain in the elderly, the prevalence and impacts of chronic pain in the elderly remain underestimated. Pain is always a recognized feature of old age to both physicians and caregivers [18]. In terms of the elderly, nociception may change with aging, and the affected elderly become accustomed to living with chronic pain, causing them not to report their pain or seek medical attention [19]. Some elderly populations suffering from moderate to severe pain reported no pain management [20, 21], and some have concerned about using analgesics [22], resulting in misuse of potentially helpful treatments [23]. Fortunately, one-third of older peoples could use both pharmacologic strategies and nonpharmacologic strategies to manage pain. The most prevalent nonpharmacologic strategies among them were exercise, nutritional supplements, ointments, heat and massage [20]. Nevertheless, there were substantial ethnic disparities reported in pain prevalence, conditions and coping strategies [24]. It is necessary to gain insight into the prevalence and pain-related health-seeking behaviors in the elderly according to different cultural and economic backgrounds.

Chronic pain is also a major health problem in China as the country possesses the largest elderly population in the world. According to the National Bureau of Statistics of China, the proportion of people aged 60 years and above had reached 16% in 2015 [25]. Thus, researchers have conducted studies to investigate the prevalence of chronic pain in the Chinese elderly population. Xue, Chu [26] investigated the prevalence of chronic pain in the elderly in those aged 80 years and above in four provinces in China, and found that 76.4% of participants suffered from chronic pain. Moreover, Wang, Xu [27] conducted a survey in elderly inpatients and reported that 55.5% of elderly inpatients had chronic pain. Accordingly, studies focusing on community-dwelling elderly mainly reported the prevalence of chronic pain rather than on characteristics like interference with daily life, health-seeking behaviors or conditions of medication use among the corresponding elderly [6, 28]. The aforementioned studies focused on specific subpopulations or prevalence of chronic pain that had limited insights into chronic pain. Therefore, getting a deeper insight into chronic pain, including its prevalence, characteristics, pain-related health-seeking behaviors in community-dwelling elderly are crucial for policymaking and chronic pain management.

### Objectives

This study aimed to investigate the prevalence, characteristics and pain-related health-seeking behaviors in the Chinese community-dwelling elderly.

## Methods

### Study design

This is a cross-sectional study that followed the Strengthening the Reporting of Observational Studies in Epidemiology (STROBE) statement.

### Setting

This study was conducted in the Sichuan Province in west China from Dec 2018 to May 2019, where people aged 60 years old or above has exceeded 9% of the population.

### Participants

The participants in this study were recruited in two stages. First, from 21 cities/autonomous prefectures in Sichuan Province, seven cities/autonomous prefectures were selected according to their socioeconomic status (Chengdu City, Luzhou City, Zigong City, Neijiang City, Nanchong City, Ganzi Autonomous Prefecture and Mianyang City). Second, a random cluster sampling method was used to recruit participants from 7 communities from the selected cities/autonomous prefectures. The inclusion criteria were: participants who were older than 60-year-old and those who agree to participate in the study. The exclusion criteria were: difficulties in communication and psychological diseases. The expected prevalence of chronic pain was set as 49.8%, according to a recent study in China [6]. A sample size of 1152 was calculated using the formula: ${Z}_{\alpha /2}^2p\left(1-p\right) DEFF/{d}^2$, in which *α* = 0.05, *p* = 0.498, DEFF = 3, *d* = 0.05 [29]. In order to compensate for potential mistakes and missing values (10%), the final sample size was 1267 participants. This study was approved by the Ethic Committee of Chengdu University.

### Data collection

Seven trained research assistants responsible for each community completed the face-to-face interviews. First, the research assistants explained the purpose and procedure of the study and obtained the participants’ written informed consent. Then, the research assistants conducted an investigation for about 30 min in a quiet room in the community hospital by the questionnaire developed for this study (Additional file 1) to collect socio-demographic data (age, sex, education level, living status, marital status, monthly income and comorbidities), characteristics of chronic pain (pain location, intensity, interference with daily life and precipitating factors), health-seeking behaviors (usage of medication and non-drug therapy) and self-rated health. The completeness of the collected data was checked by the authors in charge of data collection after research assistants completed the interviews.

### Measurements

#### Chronic pain

Pain lasting ≥3 months was defined as chronic pain following the IASP classification. Therefore, in this study, chronic pain was measured by two questions: (1) “Did you have a pain experience?”; (2) “If yes, how long did you get the pain?”. Moreover, if participants experienced chronic pain, the pain location (eight locations based on a study from Mccarthy, Bigal [30]), precipitating factors of pain were also questioned and recorded. Additionally, the use of medication and non-drug therapy was assessed by three main questions followed by specific questions: (1) When you have pain, what do you usually do? (2) Do you take any medicine to relieve the pain? (3) Have you taken any other measures to relieve the pain besides medication?

#### Pain intensity

Brief Pain Inventory (BPI) [31] scale was used to evaluate pain intensity. This subscale is recommended for elderly pain assessment as it evaluates overall pain rather than site-specific pain [32]. Participants were asked to rate the severity of pain in the last week based on four aspects (worst pain, least pain, pain on average and pain right now), according to an 11-point numeric rating scale. The “0” indicated “no pain” while “10” indicated “severe or excruciating pain you cannot imagine”. A higher score signified more severe pain. This subscale has good internal consistency with a Cronbach’s alpha coefficient of 0.84 [33].

#### Interference of pain with daily life

In this study, interference of pain with daily life was evaluated using a single pain interference subscale extracted from BPI. This pain interference subscale consisted of seven items, including general activity, mood, walk, working, relationship, sleep and enjoyment. Participants were asked to rate the degree of interference with daily life using an 11-point numeric rating scale, with “0” indicating “no interference” and “10” indicating “interference cannot tolerate”. The pain interference subscale also had good internal consistency with a Cronbach’s alpha coefficient of 0.94 [33].

#### Self-rated health

The self-rated health was evaluated by the question: “How satisfied you are with your health?” with the answers ranging from “very bad”, “bad”, “general”, “good” and “very good” [34].

### Data analysis

Chi-square test was used to compare the differences between socio-demographic groups of participants with or without chronic pain, the characteristics of chronic pain according to age and sex, and the health seeking behaviors and medication use of participants in different residence location.

Binary logistic regression was used to analyze the influence of socio-demographic factors on the presence of chronic pain.

Mann-Whitney U test was used to compare the characteristics of chronic pain according to age and sex while Student-Newman-Keuls test and Bonferroni correction method were used to compare groups in pairs where three or more groups were compared.

ANOVA and Kruskal-Wallis H test was used to compare the impact of chronic pain on daily life and self-rated health according to age.

All data analyses were performed using IBM SPSS Statistics Version 23.0 for Windows 7 (Armonk, NY: IBM Corp).

## Results

### Socio-demographic data and prevalence of chronic pain of participants

A total of 1450 elderly individuals satisfied the inclusion criteria, and 1403 of them agreed to participate in this study. Finally, 22 did not complete the interview, and 1381 participants were finally enrolled. The participants mainly consisted of females (55.3%) and elderly aged from 60 to 69 years old (45.1%). The elderly participants with no education were 26.1% of all participants. Most participants were located in urban areas (71.7%) and suffered from comorbidities (92.5%). Over 10% of participants had a monthly disposable personal income of under 275 RMB. Table 1 shows the detailed data of participants with and without chronic pain. Among all samples, 791 participants suffered from chronic pain (57.3%). Participants living in rural areas, having lower monthly income and comorbidities were more likely to have chronic pain (*p* < 0.01). However, Table 2 shows the impact of residence location on chronic pain may be due to interaction of some other factors not covered in this study, as it was not statistically significant in the binary logistic regression models.

**Table 1:** Socio-demographic data of participants with or without chronic pain (*N* = 1381)

Characteristics	Total (*N* = 1381)	Chronic pain (*N* = 791)	No chronic pain (*N* = 590)	χ^2^/Z	*P* value
Sex					
Male	617 (44.7)	339 (42.9)	278 (47.1)	2.483	0.115
Female	764 (55.3)	452 (57.1)	319 (52.9)		
Age					
60–69	623 (45.1)	357 (45.1)	266 (45.1)	− 0.420	0.675
70–79	541 (39.2)	302 (38.2)	239 (40.5)		
≥ 80	217 (15.7)	132 (16.7)	85 (14.4)		
Marital status					
Married	1023 (74.1)	594 (75.1)	429 (72.1)	3.104	0.212
Divorced/Widowed	256 (18.5)	147 (18.6)	109 (18.5)		
Unmarried	102 (7.4)	50 (6.3)	52 (8.8)		
Education level					
Illiteracy	360 (26.1)	225 (28.4)	135 (22.9)	5.502	0.064
Primary	494 (35.8)	276 (34.9)	218 (36.9)		
Secondary or above	527 (38.1)	290 (36.7)	237 (40.2)		
Residence location					
Urban area	990 (71.7)	527 (66.6)	463 (78.5)	23.380	< 0.001
Rural area	391 (28.3)	264 (33.4)	127 (21.5)		
Living alone					
Yes	129 (9.3)	69 (8.7)	60 (10.2)	0.835	0.361
No	1252 (90.7)	722 (91.3)	530 (89.8)		
Monthly DPI (RMB)					
< 275	188 (13.6)	139 (17.6)	49 (8.3)	−6.691	< 0.001
275–1700	449 (32.5)	283 (35.8)	166 (28.1)		
≥ 1700	744 (53.9)	369 (46.6)	375 (63.6)		
Comorbidity					
Yes	1278 (92.5)	765 (96.7)	513 (86.9)	46.676	< 0.001
No	103 (7.5)-	26 (3.3)	77 (13.1)		

*DPI* disposable personal income

**Table 2:** The influence of socio-demographic factors on the presence of chronic pain (*N* = 1381)

	B	S.E.	Wald	P	OR (OR95%CI)
Residence location	0.217	0.146	2.210	0.137	1.24 (0.933, 1.655)
Monthly DPI (RMB)					
275–1700 VS. < 275	0.894	0.201	19.692	0.000 ^**^	2.44 (1.647, 3.628)
≥ 1700 VS. < 275	0.411	0.135	9.347	0.002 ^**^	1.51 (1.159, 1.965)
Comorbidity	1.386	0.237	34.200	0.000 ^**^	4.00 (2.513, 6.364)
Constant	−1.515	0.277	29.956	0.000 ^**^	0.22

Assignment of variables in the model: chronic pain (Chronic pain = 1, No chronic pain = 0); Residence location (Urban area = 1, Rural area = 2); Monthly DPI (< 275 = 1, 275–1700 = 2, ≥1700 = 3); Comorbidity (Yes = 1, No = 0); R^2^ in the model: 0.084; **: *p* < 0.01

### Characteristics of chronic pain

Table 3 displays the pain intensity and pain location of participants with chronic pain. There were no significant differences in pain intensity between male and female participants regardless of worst pain, least pain, pain on average and pain right now (*p* > 0.05). Participants in the 70–79 age group had significantly higher worst pain compared to 60–69 and ≥ 80 age groups (*p* < 0.05). Participants in the 60–69 age group had significantly lower least pain and pain on average compared to the other two groups (*p* < 0.05). Participants in the 60–69 age group had significantly lower pain right now than the 70–79 group (*p* < 0.05). The most common pain locations were found to be in the legs/feet (53.3%), head (23.6%) and abdomen/pelvis (21.1%). Females had a significantly higher incidence of neck/shoulder and legs/feet pain than males (22.6% vs 14.5%, *p* < 0.01; 58.8% vs 48.7%, *p* < 0.01). Participants in the ≥80 age group significantly reported less neck/shoulder pain than the 60–69 and 70–79 age groups (*p* < 0.05). Furthermore, participants in the 70–70 age group had significantly more legs/feet pain (*p* < 0.05).

**Table 3:** Characteristics of chronic pain according to age and sex (*N* = 791)

Characteristics	Total	Male (*N* = 339)	Female (*N* = 452)	60–69 (*N* = 357)	70–79 (*N* = 302)	≥80 (*N* = 132)
Pain intensity						
Worst pain	5.6 ± 2.2	5.6 ± 2.2	5.5 ± 2.2	5.2 ± 2.2	6.0 ± 2.2	5.6 ± 2.3^**,a^
Least pain	2.2 ± 1.8	2.2 ± 1.9	2.2 ± 1.7	1.9 ± 1.6	2.5 ± 2.0	2.3 ± 1.8^**,a^
Pain on average	3.7 ± 1.7	3.7 ± 1.8	3.6 ± 1.7	3.3 ± 1.5	4.2 ± 1.9	3.9 ± 1.7^**,a^
Pain right now	2.9 ± 2.0	2.9 ± 2.1	3.0 ± 1.9	2.7 ± 1.8	3.2 ± 2.1	2.9 ± 2.0^**,a^
Pain location						
Head	187 (23.6)	85 (25.1)	102 (22.6)	91 (25.5)	72 (23.8)	24 (18.2)
Face	26 (3.3)	15 (4.4)	11 (2.4)	8 (2.2)	12 (4.0)	6 (4.5)
Neck/shoulder	151 (19.1)	49 (14.5)	102 (22.6) ^**^	80 (22.4)	59 (19.5)	12 (9.1)^**,b^
Back	114 (14.4)	42 (12.4)	72 (15.9)	42 (11.8)	49 (16.2)	23 (17.4)
Arms/hands	149 (18.8)	55 (16.2)	94 (20.8)	71 (19.9)	63 (20.9)	15 (11.4)
Legs/feet	431 (53.5)	165 (48.7)	266 (58.8) ^**^	177 (49.6)	182 (60.3)	72 (54.5)^**,b^
Chest	124 (15.7)	62 (18.3)	62 (13.7)	57 (16.0)	45 (14.9)	22 (16.7)
Abdomen/pelvis	167 (21.1)	82 (24.2)	85 (18.8)	80 (22.4)	62 (20.5)	25 (18.9)

^a^ Student-Newman-Keuls; ^b^ Bonferroni correction; ^**:^ *p* < 0.01; ^*:^ *p* < 0.05;

### Impact of chronic pain on daily and self-rated health

Table 4 illustrates interference with the daily life of chronic pain and self-rated health in participants with chronic pain. The most affected aspects of daily life were sleep (3.9 ± 2.6), general activity (3.7 ± 2.4) and walking (3.6 ± 2.6). Participants in the 60–69 age group had significantly lower general activity, mood, walking, relationship and sleep scores than the other two groups (*p* < 0.05). Moreover, participants in the 70–79 age group had significantly higher working scores compared to the other two groups (*p* < 0.05). Participants in the 70–79 age group had significantly higher enjoy scores compared to the 60–69 age group (*p* < 0.05). A total of 234 (29.6%) participants reported bad health conditions. Additionally, participants with bad health were found to increase with aging.

**Table 4:** Impact of chronic pain on daily life and self-rated health according to age

Items	Total	60–69 (*N* = 357)	70–79 (*N* = 302)	≥80 (*N* = 132)	*F/H*	*P* value
Daily activity						
General activity	3.7 ± 2.4	3.3 ± 2.2	4.2 ± 2.5	3.9 ± 2.5	25.793^a^	< 0.001
Mood	3.5 ± 2.4	3.0 ± 2.1	3.9 ± 2.5	3.6 ± 2.3	26.907^a^	< 0.001
Walk	3.6 ± 2.6	3.0 ± 2.3	4.1 ± 2.7	4.3 ± 2.8	40.149^a^	< 0.001
Working	1.4 ± 2.2	1.2 ± 1.9	1.8 ± 2.5	1.2 ± 2.1	9.014^a^	0.011
Relationships	2.7 ± 2.3	2.3 ± 2.1	3.1 ± 2.6	2.9 ± 2.4	14.579^a^	0.001
Sleep	3.9 ± 2.6	3.6 ± 2.5	4.3 ± 2.7	4.1 ± 2.7	6.919^a^	0.001
Enjoy	3.0 ± 2.4	2.6 ± 2.1	3.4 ± 2.5	3.0 ± 2.4	15.371^a^	0.001
Self-rated heath						
Very bad	33 (4.2)	11 (3.1)	13 (4.3)	9 (6.8)	17.265	< 0.001
Bad	201 (25.4)	67 (18.8)	90 (29.8)	44 (33.3)		
General	331 (41.8)	161 (45.1)	123 (40.7)	47 (35.6)		
Good	91 (11.5)	48 (13.4)	28 (9.3)	15 (11.4)		
Very good	135 (17.1)	70 (19.6)	48 (15.9)	17 (12.9)		

^a^: Student-Newman-Keuls

### Excepted precipitating factors for chronic pain

The excepted precipitating factors for chronic pain are displayed in Table 5. Up to 35.5% of chronic pain was precipitated by chills, while 32.1% of chronic pain was precipitated by excessive fatigue. Humidity was also a common precipitating factor for chronic pain (19.7%). Moreover, over one-third (37.3%) of chronic pain was precipitated by unspecific factors.

**Table 5:** Excepted precipitating factors of chronic pain (*N* = 791)

Factors	Frequency	Percentage
Excessive fatigue	254	32.1
Chill	281	35.5
Humidity	156	19.7
Life event	39	4.9
Bad mood	44	5.6
Unspecific factors	295	37.3

### Health seeking behaviors of participants with chronic pain

Table 6 displays the health-seeking behaviors of participants with chronic pain. 29.4% of participants had asked for medical help, and mostly (44.4%) chose to handle the pain themselves. Moreover, up to 40.8% of participants with chronic pain did not receive medication. The medication use rate in rural areas was significantly higher than in urban areas (*p* < 0.001). The most popular non-drug therapies adopted were massages (21.4%), hot/cold compresses (16.4%) and acupuncture (13.8%). Those living in urban areas significantly intended to take acupuncture, cupping therapy, electrical stimulation and massage to cope with chronic pain than the participants in a rural area.

**Table 6:** Health seeking behaviors and medication use of participants with chronic pain (*N* = 791)

Items	Total	Residence location		χ^2^	*P* value
		Urban (*N* = 527)	Rural (*N* = 264)		
Health seeking					
Enduring	207 (26.2)	138 (26.2)	69 (26.1)	1.485	0.467
Handling by oneself	351 (44.4)	227 (43.1)	124 (47.0)		
Seeking for medical help	233 (29.4)	162 (30.7)	71 (26.9)		
Medication use					
Yes	468 (59.2)	281 (53.3)	187 (70.8)	22.308	< 0.001
No	323 (40.8)	246 (46.7)	77 (29.2)		
Non-drug therapy adopted					
No	319 (40.3)	200 (38.0)	119 (45.1)	3.711	0.054
Yes	472 (59.7)	327 (62.0)	145 (54.9)		
Acupuncture	109 (13.8)	84 (15.9)	25 (9.5)	6.196	0.013
Cupping therapy	71 (9.0)	55 (10.4)	16 (6.1)	4.122	0.042
Electrical stimulation	48 (6.1)	46 (8.7)	2 (0.8)	19.606	< 0.001
Massage	169 (21.4)	133 (25.2)	36 (13.6)	14.089	< 0.001
Distraction	43 (5.4)	33 (6.3)	10 (3.8)	2.094	0.148
Hot/cold compress	130 (16.4)	80 (15.1)	50 (18.9)	1.810	0.179
Others	31 (3.9)	25 (4.7)	6 (2.3)	2.852	0.091

## Discussion

In this cross-sectional study, we learned the basic characteristics of chronic pain in the elderly community in Sichuan Province. 57.3% of community-dwelling older adults residing in west China were found to have chronic pain, precipitated mainly by chills and excessive fatigue. In addition, elderly individuals aged 60–69 years were more likely to have mild pain. The first three common pain locations were observed in the legs/feet, head and abdomen/pelvis. The most affected aspect of daily life due to chronic pain was sleep. Moreover, elderly aged between 60 and 69 years old were less affected by chronic pain according to general activity, mood, walk, relationship and sleep. Nearly half of those with chronic pain did not use medication and over half adopted non-drug therapy.

Surprisingly, the prevalence of chronic pain in this study was significantly higher than previous studies conducted by Li, Chen [6] and Si, Wang [28], which respectively reported that the prevalence of chronic pain in Chinese community-dwelling elderly were 49.8 and 41.1%. The reasons why the prevalence was higher in this study may be the gap of economic and medical resources between East China and West China. Li, Chen [6] and Si, Wang [28] both recruited participants from East China whose economic and medical resources are much better than that of west China, and economic status was previously observed to influence the incidence of chronic pain [35]. Moreover, Si, Wang [28] only investigated samples from the capital city owning the best economic and medical resources in Shandong Province, leading to a lower incidence of chronic pain.

In terms of sex, most previous studies revealed that females were more likely to have chronic pain [8, 9, 28, 36], which was inconsistent with this study. It is generally believed that females are more sensitive to pain due to their unique biological or psychological mechanisms [37, 38]. Moreover, females usually live longer than males; hence, the difference increases with aging. In this respect, this study did not find any differences in the prevalence of chronic pain based on sex, suggesting that regional and cultural differences may need to be considered when the relationship between sex and chronic pain be examined [39, 40].

The current study results showed that pain intensity did not increase with aging and decreased after 80 years of age. However, other studies have also found a decrease in pain prevalence with age up to 85 years [41, 42], which may be related to the decreased perception of pain caused by sensory dysfunction in people over 80 years old.

This study found that elderly living with lower monthly incomes had a higher prevalence of chronic pain, confirmed by other studies [4, 43, 44]. Socioeconomic factors have been associated with worse health outcomes, for those living in poverty, low incomes haunt each financial decision, and many are unable to consistently afford prescribed interventions such as medications and ongoing visits to health care providers to manage their health [45]. Therefore, it is important for policymakers to pay more attention to the elderly population.

In our study, significant relationship between the prevalence of chronic pain and education level was not observed, which was inconsistent with previous studies that reported a lower level of education indicated a higher incidence of chronic pain [28, 36]. They believed that patients with low education level might delay the visit or treatment due to insufficient health awareness, who fail to treat chronic pain-related illnesses early [46]. Therefore, the results of this study may imply that older people with higher education in western China still lack sufficient health awareness. However, another factor was that a higher prevalence of chronic pain was observed in elderly with a lower level of education, which may be associated with the wrong perception that chronic pain is due to low education levels rather than low socioeconomic status [36].

This study found that elderly living in rural areas had a lower prevalence of chronic pain, but the impact of residence location on chronic pain may be due to interaction of some other factors, which might be lifestyle, economic burden and health seeking behavior. The economic conditions in urban areas are better than those in rural areas in western China, however earlier findings have shown that older people living in poorer neighborhoods are more likely to suffer from chronic pain [45, 47]. The reason for this paradoxical result in this study might be because older people living in cities have a modern way of life, which is not healthier and leading to increased risk for kinds of chronic disease, including chronic pain [48]. Studies have shown that the health care costs was higher in developed cities [49], which may lead to the lack of treatment for pain due to the heavy economic burden of disease treatment in some elderly people. In this study it was also found that the medication use rate in rural areas was higher than in urban areas among older adults with chronic pain.

In this study, the most common pain locations were found in the legs/feet, head and abdomen/pelvis. However, the ranking of the reported pain locations was observed to vary greatly across different studies. For example, Korean elderly individuals most frequently reported back pain [50]. Moreover, the elderly from the UK and Spain mostly reported lower limb pain [51, 52], while the Polish elderly mainly suffered from pain in their lumbar regions [18].

In daily life activities, chronic pain was found to interfere mainly in sleep, general activity and walk in this study. Si, Wang [28] also found a strong association between sleep disturbance, decreased physical activity and chronic pain in the elderly, which may be due to functional changes in the nervous system, where pain and sleep are both modulated due to long-term chronic pain [53]. It was previously found to be necessary to focus on the sleep quality of elderly with chronic pain. In terms of activity, fear of pain avoid them from exercise, daily self-care, and even any move [54]., which could endanger their independence and quality of life, with reduced levels of fitness and function leading to increased levels of disability [10]. Therefore, it is important for health care providers to educate older people to maintain and increase physical activities.

Unsurprisingly, only 29.4% of participants in this study actively sought medical help, and over 40% did not receive medication. The corresponding result was similar to that of Liberman, Freud [55], who reported that only 41.1% of the elderly used medication. However, over half of participants in this study adopted non-drug therapies such as massages, hot/cold compresses, and acupuncture, which may have reduced the medication use rate, especially in elderly living in urban areas. Thus, providing easy access to medication assistance and scientific non-drug therapy guidance to the elderly suffering from chronic pain may benefit and improve pain management.

There are some limitations to this study. First, this was a cross-sectional study conducted in west China, where economic and medical statuses differed from other parts of China. Thus, the representativeness of the sample was limited because economic and medical resources influenced the prevalence and characteristics of chronic pain. Second, precipitating factors and medication use for chronic pain may vary according to the different biological or pathological characteristics of chronic pain. In this study, we could not verify these variations; hence, researchers should be cautious in generalizing the results of the precipitating factors, pain locations and medication use conditions.

## Conclusion

Chronic pain is a common health concern in the Chinese community-dwelling elderly with a prevalence of 57.3%. The prevalence and intensity of chronic pain did not increase with aging and showed no differences with respect to sex. Pain in the legs/feet was the most reported pain location in both males and females. Furthermore, easier access to medication assistance and scientific guidance for non-drug therapy may be helpful in improving pain management in the elderly population.

## Supplementary Information

**Additional file 1.**

## Data Availability

The data that supports the findings in this study can be made available through contacting the corresponding author under reasonable request.

## Abbreviations

IASP: International Association for the Study of Pain
STROBE: Strengthening the Reporting of Observational Studies in Epidemiology
BPI: Brief Pain Inventory
RMB: Renminbi
DPI: Disposable personal income
UK: United Kingdom
