# Supplementary material for: Prevalence and characteristics of chronic Pain in the Chinese community-dwelling elderly: a cross-sectional study
Source: BMC Geriatr. 2021 Oct 7;21:534. doi: 10.1186/s12877-021-02432-2 (PMC8499479; doi:10.1186/s12877-021-02432-2)
Supplement: Supplementary file 1 — Additional file 1. [file 12877_2021_2432_MOESM1_ESM.docx]

Additional file 1

**A Questionnaire of Chronic Pain in the Elderly in Sichuan Province**

Dear old friends:

Thank you very much for your time. We are conducting a research about chronic pain in the elderly, hope to better understand their conditions about chronic pain. The information you provide has a great significance to solving the problem of chronic pain management. The questions have no set answer, you can finish it depending on your actual situation.

Thank you for your cooperation!

**General information**

1. Your sex: ○Male ○Female

2. Your age: _________

3. Are you in marriage?

○Yes ○No (specific situation: ○Divorced/widowed ○Unmarried)

4. Your education condition:

○Illiterate ○Primary school ○High school ○College degree or above

5. Your nationality:

○Han ○Specifically: ____________

6. Do you have any religious belief?

○No ○Yes, it is ____________

7. Your residence is in:

○Urban area ○Rural area

8. Your living situation:

○Living alone with your spouse

○Living with your children

○Living in a nursing home with your spouse and children

9. What is the per capita income of your family every month?

○Less than 275 RMB

○275 ~ 1700 RMB

○More than 1700 RMB

10. Do you have health insurance? (Multiple options available)

○Basic Medical Insurance for Urban Employees (Social Security)

○New rural cooperative medical care (new rural cooperative medical care)

○Commercial medical insurance

11. What chronic diseases do you suffer from?

○Hypertension

○Coronary heart disease

○Chronic heart failure

○Hyperlipidemia diabetes

○Gout stroke (stroke)

○Pulmonary heart disease

○Chronic obstruction

○Chronic fulcrum of lung

○Asthma

○Osteoarthritis,

○Rheumatism,

○Osteoporosis,

○Cervical/lumbar spine disease

○Cirrhosis

○Hepatitis

○Gallstones/cholecystitis

○Chronic gastritis peptic ulcer

○Chronic renal failure

○Chronic nephritis urinary stone

○Cancer

○Others: ______________

12. How satisfied you are with your health:

○Very bad ○Bad ○General ○Good ○Very good

**Chronic pain**

This questionnaire is used to know your current chronic pain situation, please choose according to the actual situation.

Did you have a pain experience？

○No

○Yes (Answer questions 1)

1. how long did you get the pain？

○Less than a mouth

○One to three months

○More than three months (Answer questions 2-10 below)

2. Where the pain occurs in your body (multiple options)

○Head

○Face

○Arm/Hands

○Legs/Feet

○Neck/Shoulder

○Back

○Chest

○Abdomen/Pelvis

3. How often do these pains occur?

○Every _____________ occurs

○Not clear or irregular

4. Do you have any of the following precipitating factors before the onset of your pain? (Multiple options)

○Excessive fatigue

○Chill

○Humidity

○Life event

○Bad mood

○Unspecific factors

5. According to the description of the pain in the picture below, and in questions (1) to (4) below, choose a number from the numbers 0 to 10 to indicate the degree of your pain. The higher the number, the more painful it is.


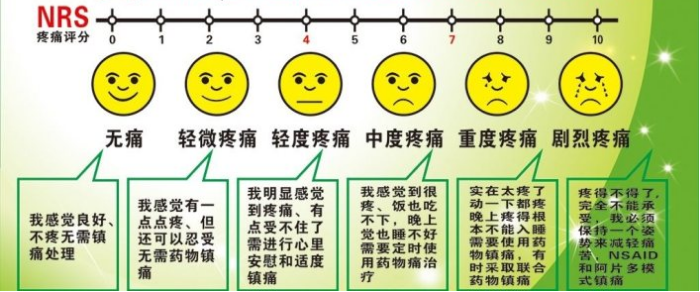


1. The most severe pain you have experienced is:

0 1 2 3 4 5 6 7 8 9 10

1. The least pain you have experienced is

0 1 2 3 4 5 6 7 8 9 10

1. The amount of pain you normally experience is:

0 1 2 3 4 5 6 7 8 9 10

1. The degree of your pain is:

0 1 2 3 4 5 6 7 8 9 10

6. Please circle a number from 0 to 10 to indicate how much your pain affects the following conditions. The higher the number, the greater the impact. (A scale of 0 means no impact, 1-3 means small, 4-6 means moderate, 7-9 means large, and 10 means very large)

(1) General activities (such as washing, doing housework, etc.)

0 1 2 3 4 5 7 8 9 10

(2) Mood

0 1 2 3 4 5 6 7 8 9 10

(3) Walking ability

0 1 2 3 4 5 6 7 8 9 10

(4) Daily work (retired or unemployed at home)

0 1 2 3 4 5 6 7 8 9 10

(5) Relationships with others (e.g., get-togethers with friends, family)

0 1 2 3 4 5 6 7 8 9 10

(6) Sleep

0 1 2 3 4 5 6 7 8 9 10

(7) Enjoy (e.g., hobbies, recreation, etc.)

0 1 2 3 4 5 6 7 8 9 10

7. Do you think these pains occur because :(multiple choice)

○Treatment

○Diseases

○injury (trauma)

○Aging

○Bad habits, e.g. _______________________)

○Others, such as ________________________

8. When you have pain, what do you usually do

○Do nothing

○Handle it myself (please answer the following ① questions)

① The most common method you use is:

○Eat painkiller by oneself

○Hot compress or cold compress

○Plasters

○Massage

○Others, such as __________

○Go to the doctor (Please answer the following ②③ questions)

② The most common medical institutions you visit are:

○Hospitals

○Community

○Western medicine clinics

○Chinese medicine clinics

③ The department you visit most often is:

○Pain department

○Orthopedics department

○Rheumatology department

○Rehabilitation department

○Traditional Chinese medicine department

○Other ______

9. Do you take medicine when pain occurs?

○Take medicine (please answer the following questions ① ② ③ ④)

① The source of the medicine:

○Prescription drug

○Self-purchased medications

② How do you take the medicine?

○Take it according to your doctor's instructions.

○Take it according to your experience

③ How much did the medicine relieve your pain?

○No remission

○10%

○20%

○30%

○40%

○50%

○60%

○70%

○80%

○90%

○100%

④ The name of the painkiller you used

○ ___________________________

○I don't know

○Don't take medicine (Answer ⑤)

⑤ The reason why you do not take medicine is (multiple options) :

○The effect is not good,

○It is not necessary to take,

○Afraid of addiction,

○Afraid of side effects

○High cost

○The doctor did not prescribe medicine to buy them

○Other _______________)

10. Have you taken any other measures to relieve the pain besides medication?

○Yes (Please answer the following ① ② questions)

① Which of the pain relief measures you have adopted are more effective for pain relief? (Multiple options)

○Acupuncture

○Cupping

○Electrotherapy

○Massage

○Hot compress or cold compress

○Plaster

○Patting

○Distraction (listening to music, looking for something to do, etc.)

○Others: ___________

② How much did these measures relieve your pain ?

○No remission

○10%

○20%

○30%

○40%

○50%

○60%

○70%

○80%

○90%

○100%

○No (Please answer question ③ below)

③ What's the reason why you didn't take other measures to relieve the pain? (Multiple options)

○Effect is not good feel

○Not necessary

○Do not know which measures are effective

○Other ______________
